# Cryo-EM structures of human calcium homeostasis modulator 5

**DOI:** 10.1038/s41421-020-00228-z

**Published:** 2020-11-10

**Authors:** Jie Liu, Futang Wan, Qiuheng Jin, Xiaoxiao Li, Eijaz Ahmed Bhat, Jiangtao Guo, Ming Lei, Fenghui Guan, Jian Wu, Sheng Ye

**Affiliations:** 1 Life Sciences Institute, Zhejiang University, Hangzhou, Zhejiang 310058 China; 2 Ninth People’s Hospital, Shanghai Jiao Tong University School of Medicine, Shanghai 200125, China; 3 Shanghai Institute of Precision Medicine, Shanghai 200125, China; 4 Department of Biophysics, Department of Pathology of Sir Run Run Shaw Hospital, Zhejiang University School of Medicine, Hangzhou, Zhejiang 310058 China; 5 Department of Biophysics, Institute of Neuroscience, NHC and CAMS Key Laboratory of Medical Neurobiology, Zhejiang University School of Medicine, Hangzhou, Zhejiang 310058 China; 6 State Key Laboratory of Oncogenes and Related Genes, Shanghai Jiao Tong University School of Medicine, Shanghai 200125, China; 7 Key laboratory of Cell Differentiation and Apoptosis of Chinese Ministry of Education, Shanghai Jiao Tong University School of Medicine, Shanghai 200025, China; 8 Tianjin Key Laboratory of Function and Application of Biological Macromolecular Structures, School of Life Sciences, Tianjin University, 92 Weijin Road, Nankai District, Tianjin 300072, China; 9 Shanghai Key Laboratory of Translational Medicine on Ear and Nose diseases, Shanghai 200125, China

**Keywords:** Electron microscopy, Protein transport

Dear Editor,

Calcium homeostasis modulators (CALHMs) are the most recently identified large pore ATP-releasing channels, playing important roles in neuronal functions including gustatory signaling^1,2^ and neuronal excitability^3^. Dysfunction of CALHMs has been linked to pathologies of depression^4^ and Alzheimer’s disease^5^. Therefore, CALHM family proteins have received increasing attention in recent neurobiological studies^6,7^. *CALHM* genes are present throughout vertebrates^5^, and six CALHM homologs have been identified in humans that share an overall 20%–50% sequence similarity^8^.

The most extensively studied CALHM1 is an ATP- and ion-permeable channel that is activated by membrane depolarization or removal of extracellular Ca^2+^^5,8^. It was suggested that the N-terminal helix or the second extracellular loop of CALHM1 may play a role in voltage-dependent gating^9^. Despite having been investigated by several studies, the activation mechanism of CALHM2 remains controversial. Mostly, CALHM2 was considered insensitive to voltage changes or removal of extracellular Ca^2+^^1,10^. Similarly, it was reported that CALHM3, 4, 6 are either not forming functional homomeric channel or activated by unknown stimulations^11^.

Recent progress in structural characterization of CALHMs explicated multiple oligomeric assemblies of channels and different conformations of pore architecture, yet the gating mechanism of CALHMs is still obscure. To further investigate the CALHM channels, we purified human CALHM5 and determined its structure by cryo-electron microscopy (cryo-EM). For CALHM5 expression, we infected the HEK293S cells with baculovirus generated using the BacMam system and boosted the expression level of CALHM5 with supplementary sodium butyrate. The protein was purified in the detergent and then used for data collection by cryo-EM. The 2D class averages indicated a significant heterogeneity of the channel stoichiometry, ranging from decamer to tridecamer (Fig. 1a). Due to the strong orientational preference of CALHM5 particles, the reconstructed 3D map was at a low resolution (~8 Å) (Supplementary Fig. S1). To solve this problem, we then incorporated the purified CALHM5 into covalently circularized nanodiscs before cryo-EM studies. The 2D class averages showed a different oligomeric distribution with a majority of channels being organized as undecamer (Fig. 1b). Finally, 3D classification and refinement yielded high-resolution maps bearing apparent C11 symmetry (Fig. 1c, d; Supplementary Fig. S2). The reconstructed map displays well-defined side-chain densities, allowing de novo model building for most amino acids with exceptions of residues M1–D2 and G288–M309, which are presumably disordered and thus invisible in the density map (Supplementary Fig. S3). We determined the structures of CALHM5 in the presence of EDTA, Ca^2+^ or rubidium red (RUR) at overall resolutions of 2.89 Å, 2.90 Å, and 2.64 Å, respectively (Supplementary Figs. S2, S4a, b and Table S1). No conformational differences are observed among the three structures (Supplementary Fig. S4c), and no densities corresponding to Ca^2+^ or RUR are reconstituted, indicating that CALHM5 channel may not share similar inhibition mechanisms with CALHM1^2,5^.

**Fig. 1:**
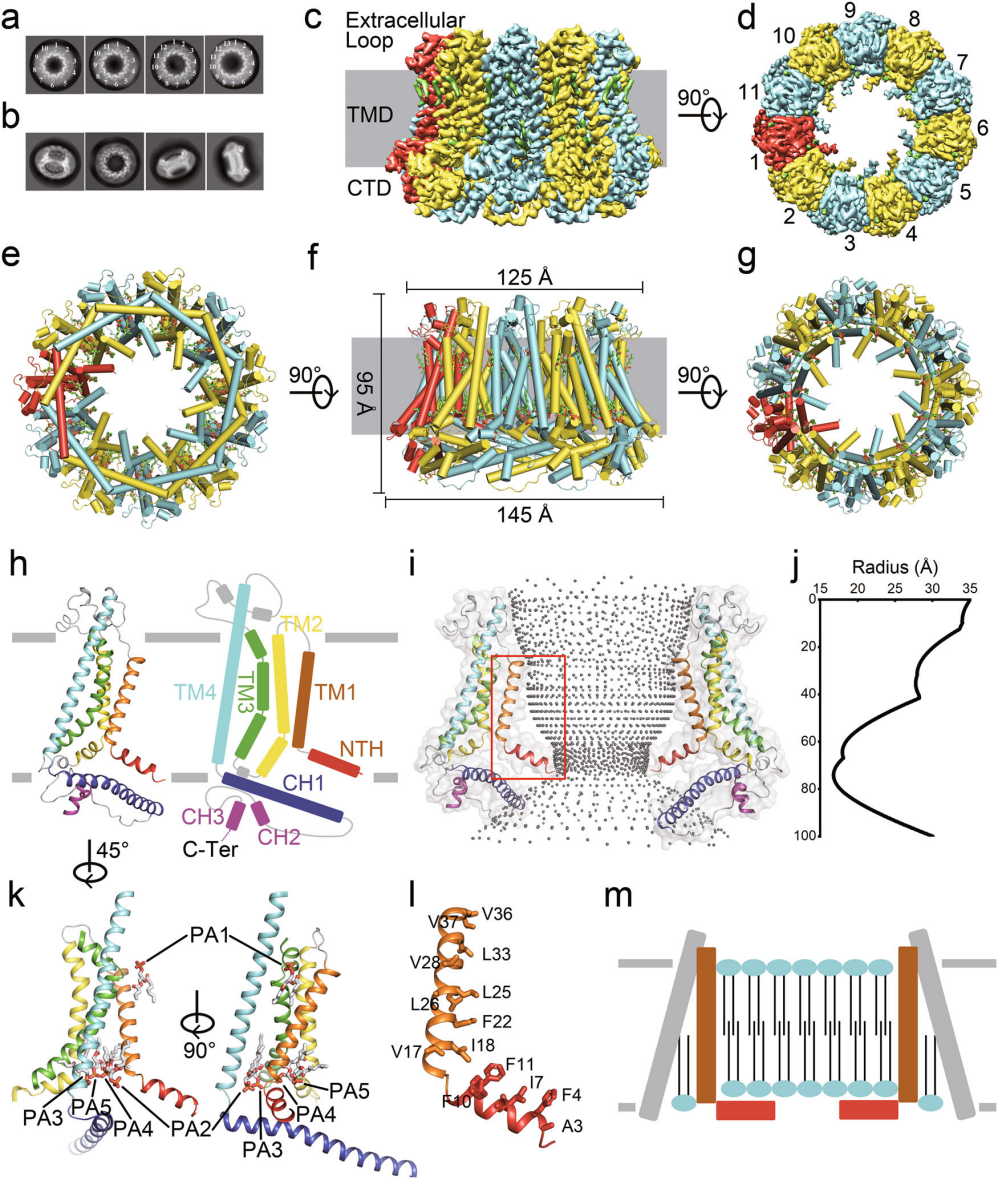
Cryo-EM structure of human CALHM5 channel. **a** Selected 2D averages of CALHM5 in detergent. **b** Selected 2D averages of CALHM5 in lipid nanodiscs. **c**, **d** The cryo-EM density map of CALHM5 viewed parallel to the plasma membrane (**c**) or from the extracellular side down the symmetry axis (**d**). **e**–**g** Cartoon representation of the CALHM5 atomic model viewed in the same direction as in **c** (**f**) or **d** (**g**), or from the cytoplasmic side up the symmetry axis (**e**). **h** Cartoon representation of the CALHM5 protomer structure and schematic representation of the CALHM5 topology. **i** Cartoon representation of the channel pore with substrate conductive path displayed in gray sticks. **j** The pore radius along the substrate conductive path calculated by HOLE program^1^. **k** Two views of cartoon representation of the CALHM5 protomer structure with 5 phospholipids highlighted in stick. **l** Zoomed-in view of the red box region in **i** with hydrophobic residues shown in stick and labeled. **m** Schematic illustration of hypothetical CALHM5 structure in vivo in the closed state.

CALHM5 assembles as an undecamer, showing an overall truncated cone-shaped architecture, with approximate dimensions of 95 Å in height spanning the cell membrane, 125 Å in extracellular width, and 145 Å in intracellular width (Fig. 1e–g). CALHM5 shares the same transmembrane topology and, similar subunit architecture with other CALHMs (Fig. 1h; Supplementary Fig. S5). Each subunit consists of a large transmembrane domain (TMD) that comprises four transmembrane helices TM1–4 and an N-terminal helix (NTH), an intracellular C-terminal domain (CTD) consisting of three α-helices CH1–3 and an extracellular loop region (Fig. 1h). The pore-lining helices TM1 and NTH were always poorly defined due to the high mobility among the known structures of CALHM members^10,12^. However, these regions are well defined in the CALHM5 structure (Supplementary Fig. S3), where TM1 is poised parallel to the central axis of symmetry and the preceding NTH projects toward the axis forming a constriction. CALHM5 exhibits an unusually large pore along the symmetric axis which is broad at both entrances with a measured diameter of ~60 Å and is slightly narrowed down at constriction (Fig. 1i, j). The atomic model of CALHM5 is determined from the third N-terminus residue, allowing us to estimate a minimum pore diameter of 34 Å based on modeling with two additional alanine residues as α-helix extending towards pore axis (Fig. 1j).

Although a previous study suggested that the “vertical conformation” of TM1 represents the open state of channel^12^, we speculate that this conformation is impermeable to ions or substances and that the channel pore is sealed by lipids. We observed extra amorphous density in the middle of the CALHM5 channel (Supplementary Fig. S6) and five lipid-like densities per CALHM5 monomer annotated as PA1–5 (Fig. 1k). PA1 is oriented upwards, with headgroups toward positively charged residues at the short extracellular loop (Supplementary Fig. S7b, c). PA2–5 are oriented in the opposite direction within a cavity formed by intracellular halves of TM2–4, where a network of hydrogen bonds and salt bridges holds these lipid phosphates in place (Supplementary Fig. S7d, e). Given that all PAs oriented with a bilayer-like configuration and the pore-lining residues are highly hydrophobic (Fig. 1l; Supplementary Fig. S8), the amorphous densities inside the pore might be corresponding to lipids forming bilayer as PAs (Fig.1m; Supplementary Fig. S9), which would impede the conduction of ions or charged substances. Such a lipid-involved gating mechanism of certain CALHM members was also proposed and testified by molecular dynamics simulation^10^.

These tightly bound lipids facilitate the stabilization of CALHM5 structure. In CALHM2 and CALHM1, TM1 from different monomers are distant from each other in the oligomer, and exclusively interacts with TM3. This loose interaction among TM1s might result in their high mobility. But in our investigated structure of CALHM5, PA1’s hydrophilic head forms hydrogen bonds with surrounding residues including Arg32 of TM1, Asn121 of TM3, and Val37 of TM1′ (apostrophe indicates the adjacent subunit), whereas the hydrophobic tails fill the gap between two adjacent TM1 helices (Supplementary Fig. S7a, b). Interestingly, we noticed that these residues are not conserved among CALHMs and some of them, such as Asn121 and Arg32 (Arg124 and Glu37 in CALHM2), are also involved in RUR binding in CALHM2 (Supplementary Fig. S10). It was reported that CALHM2 with a charge-reversing mutation E37R displays insensitivity to RUR^12^, which is confirmed with our present result that RUR may not interact with CALHM5. On the intracellular side of TMD, TM2 is kinked at Asn71 (designated as TM2a and 2b separately) and TM2b projects away from the pore axis with TM3 being bent in the same direction, generating a cleft between TM1 and other transmembrane helices. PA2–5 bridge this gap, and thus stabilize the pore architecture. Moreover, PA2 and PA3 surrounded by TM1, TM4, and TM2′ are involved in subunit–subunit interactions. All CALHM homologs share a similar subunit architecture and the cleft is a general structural feature. Recent studies have suggested that different lipid molecules could reside in this cleft, such as cholesteryl hemisuccinate in CALHM1^13^, phosphatidylcholine in CALHM4^13^ and lipid-like densities in CALHM2^12^. These results indicate that lipids might be involved in regulating the conformational changes during channel gating.

In addition to TMD, the CTD seems to be involved in lipid binding as well. Major residues at CH1 facing the cleft are polar residues. Together with marginal residues of TMD, they form a positively charged pocket which can accommodate the phosphate groups (Supplementary Figs. S7d, e, and S11). Since each CH1 interacts with adjacent CH1 helices, forming another two conserved subunit–subunit interfaces, these lipids are likely to affect all assembly interfaces in CALHM5. Structural superpositions unambiguously demonstrate that the difference between undecameric CALHM5 and octameric CALHM1 is much larger than that between undecameric CALHM5 and CALHM2, suggesting that the arrangements of TMD and CTD determine the oligomeric state of CALHM channels, which has been proposed previously^12^. In addition, we observed diverse oligomeric states of CALHM5 solubilized in detergent (Fig. 1a) and obtained a dodecameric density map (Supplementary Fig. S1), whereas channels reconstituted in nanodiscs are predominantly undecamer, indicating that lipids are related to subunits stoichiometry. Taken together, we speculate that lipids are one of the modulators regulating CALHM assembly through affecting TMD–CTD arrangement. The hypothesis was partially supported by recent studies manifesting that both CALHM4 and CALHM6 have different oligomeric states and that chimeric constructs of CTD and TMD from different CALHMs with disparate subunit stoichiometry could assemble a chimeric channel conformationally resembling native CALHMs^10,11,13^. These studies suggested that the interfaces between CALHM subunits could tolerate subtle disturbance and the oligomeric state is controlled by the arrangement of TMD and CTD^10^. These results underline the ability of CALHM proteins to constitute membrane channels with different pore sizes which are regulated by membrane lipids.

In summary, we present the cryo-EM structure of human CALHM5 reconstituted in lipid nanodiscs. Despite adopting a general overall architecture as previously determined CALHMs^10–12^, CALHM5 exhibits clearly defined vertical TM1 and intro-projecting NTH which may be stabilized upon lipid binding. In addition, CALHM5 is the first CALHM structure with the well-defined N-terminal region, demonstrating a hydrophobic and unusually large channel pore, reinforcing the previously reported lipid-involved gating mechanism. The CALHM5 structures lay the foundation and provide an opportunity for further investigation of the function and the regulation mechanisms of lipids in CALHM channel assembly, property, and gating.

## Supplementary information

Supplementary Information

## Data Availability

Structure coordinates and cryo-EM density maps of CALHM5 in the presence of EDTA, Ca^2+^ and rubidium red have been deposited in the protein data bank under accession numbers 7D61, 7D65, 7D60 and EMD-30587, EMD-30589, EMD-30586, respectively.

## Supplementary information

**Supplementary Information** accompanies the paper at (10.1038/s41421-020-00228-z).
