# Supplementary material for: Cryo-EM structures of human calcium homeostasis modulator 5
Source: Cell Discov. 2020 Nov 10;6:81. doi: 10.1038/s41421-020-00228-z (PMC7652935; doi:10.1038/s41421-020-00228-z)
Supplement: Supplementary file 1 — Supplementary Information [file 41421_2020_228_MOESM1_ESM.pdf]

Supplementary information, Data S1

Methods and Materials

Cloning, Expression and purification

The gene encoding full-length human CALHM5 (NCBI Reference Sequence: NP_714922.1) was synthesized with codon optimized for *Homo sapiens* by Genscript company and was cloned into a BacMam expression vector (pEZT). For protein purification, StrepII-tag was added at the C-terminus of the gene.

Recombinant baculovirus of CALHM5 was generated using the Bac-to-Bac system (Invitrogen) following manufacturer's instructions. Briefly, DH10Bac cells were used to generate bacmid vectors and Sf9 cells (Invitrogen) were used for bacmids transfection to generate P1 virus. P2 and P3 virus were then generated stepwise by infecting cells with the previous generation virus at a MOI of 0.05. For large-scale expression, HEK293S-GnTI^{-} cells growing in SMM293-TI medium(Sino Biological Inc.) supplemented with 2% FBS (Gibco), 2 mM GlutaMAX-I (Gibco) were infected with P3 virus at a ratio of 1:10 (virus:HEK293S, v:v) and expression level was further boosted by adding 10 mM sodium butyrate (Sigma-Aldrich) after 10 hours. Cells were cultured at 37°C, 8% CO_2 for 36 hr before harvest.

The cell pellet was resuspended lysis buffer (25 mM HEPES-NaOH pH 7.5, 150 mM NaCl, supplement with 2 μg/ml DNase I, 2 μg/ml pepstatin, 2 μg/ml leupeptin, 2 μg/ml aprotinin and 1 mM PMSF) and homogenized by sonication on ice. The membrane fraction was collected by high-speed centrifugation (10,000 g for 30 min) followed ultracentrifugation (200,000 g for 1 hr) and the pellet was mechanically homogenized and solubilized in extraction buffer (25 mM HEPES-NaOH pH7.5, 150 mM NaCl, 1% (w/v) N-Dodecyl-β-D-maltopyranoside (DDM, Anatrace) and 0.1% (w/v) cholesteryl hemisuccinate (CHS, Sigma Aldrich)) for 3 hr along with gentle agitation on ice. Solubilized CALHM5 was separated by ultracentrifugation (10, 000 g for 40 min at 4°C) and the supernatant mixed with Strep-Tactin Sepharose resin (IBA Lifesciences) pre-equilibrated with SEC buffer (25 mM HEPES-NaOH pH 7.5, 150 mM NaCl, 0.5 mM DDM) was incubated for 1

hr under gentle agitation at 4^{0}C. The resin was collected on a disposable gravity column (Bio-Rad), washed with SEC buffer for 8 column volumes and eluted with the same buffer supplement with 10 mM D-desthiobiotin (Sigma-Aldrich). CALHM5 was further purified by size-exclusion chromatography (SEC) on a Superose 6 10/300 GL column (GE Healthcare) pre-equilibrated with SEC buffer. The peak fractions were pooled, concentrated to 3 mg/ml using a spin concentrator 100-kD cutoff (Millipore) for cryo-electron microscopy analysis.

Covalently circularized nanodisc (cND) constitution

An optimized version of MSP1E3D1 (His-lsMSP1E3D1) with solubility-enhancing mutations and a C-terminal eSrt-recognition motif for circularization by SrtA was synthesized and cloned into pET28a(+). A codon-optimized version of SrtA was also synthesized and cloned into pET28a(+). His-lsMSP1E3D1 and SrtA were expressed, purified for preparing circularized MSP1ED1 (csMSP1ED1) as previously described [1].

For CALHM5 nanodiscs reconstitution, protein was purified as described above and was concentrated to 5mg/ml. Purified CALHM5 was mixed with csMSP1E3D1and soybean polar lipid extract (Avanti) at final molar ratio of 2:1:100, respectively. The mixture was then incubated end-to-end for 1hr at room temperature. Detergents were removed by adding Bio-Beads SM2 (Bio-Rad) to a concentration of 20 mg/ml with gentle agitation for 12 hr followed by Bio-Beads supplement to a final concentration of 100mg/ml. Afterwards, the sample was filtered with a disposable gravity column and reconstituted CALHM5 nanodiscs was separated on a Superose 6 10/300 GL column pre-equilibrated with 25 mM HEPES-NaOH pH 7.5, 150 mM NaCl. The peak fractions were pooled, concentrated to ~3 mg/ml for cryo-electron analysis.

Cryo-EM Sample preparation and Data Acquisition

3.0 μl of purified protein (either in detergents or reconsitutied into nanodisc) was piptted onto a glow-discharged holey carbon grids (Quantifoil R1.2/1.3 Au 200 mesh). Grids were blotted for 5.5 s with a blotting force of -1 and a humidity of 100% at 8 °C and flash frozen into liquid nitrogen cooled liquid ethane using a Mark IV

Vitrobot (Thermo Fisher). Micrographs were acquired on a Titan Krios microscope (FEI) operated at 300 kV and equipped with an K3 Summit direct electron detector camera (Gatan) set to super-resolution counting mode. EPU was used for automated data collection following standard procedure. All images were recorded with a nominal magnification of 81, 000 X, corresponding to a pixel size of 1.09Å and with a set defocus range of 1.2 ~ 1.6 μm. 32 frames were collected for each stack with an exposing time of 1.5625 s per frame with a total dose rate of 50 e-/Å2.

Cryo-EM Data Processing

Data processing for CALHM5, a total of 2571 movies were collected, aligned, and does-weighted to correct for movement during imaging and account for radiation damage via Motioncor2[2]. The CTF parameters for each micrograph were determined by Gctf[3].

In total, 2,954,203 particles were auto-picked and selected for further 2D classification, which yielded a stack of 2,395,825 particles. Then all these particles were subjected to 3D classification using a 60Å-low pass filtered initial model as a reference. The major class with reasonable features containing about 576,663 particles was applied for further 3D refinement. Since CALHM5 appears as a undecamer, the C11 symmetry was applied during 3D refinement, producing a final map at 2.6 Å resolution based on the gold-standard FSC cut-off criterion at 0.143. The density map was sharpened by applying a negative temperature factor automatically estimated by post-processing program of RELION[4]. Local resolution estimates were determined using RELION.

Model Building, Refinement and Validation

De novo atomic model building based on 2.6 Å resolution density map of CALHM5 was performed in Coot[5] and PHENIX[6]. The backbone was built by model building software phenix.map_to_model[7] with C11 symmetry information. Amino acid assignment was manually achieved in Coot, based on the clearly defined density for two pairs of disulfate bond and bulky residues (Phe, Trp, Tyr, Arg) on extracellular linker and four transmembrane helices. Models were refined against summed maps using phenix.real_space_refine[8], with secondary structure restraints

and non-crystallography symmetry applied, and re-adjusted in Coot, iteratively, until no further improvement in model geometry could be obtained. The initial EM density map allowed us to construct a CALHM5 model containing residues 3-288.

The structure comparisons between CALHM5 and other CALHMs, including human CALHM2 (PDB: 6UIV), chicken CALHM1 (PDB:6VAM) were processed using CCP4i2. All the figures of models were prepared in PyMol[9] and all the figures of density maps were prepared in UCSF Chimera[10].

References

1. Johansen, N. T. *et al.* Circularized and solubility-enhanced MSP s facilitate simple and high-yield production of stable nanodiscs for studies of membrane proteins in solution. *Febs J.* **286**, 1734–1751 (2019).

2. Zheng, S. Q. *et al.* MotionCor2: anisotropic correction of beam-induced motion for improved cryo-electron microscopy. *Nat. Methods* **14**, 331–332 (2017).

3. Zhang, K. Gctf: Real-time CTF determination and correction. *J. Struct. Biol.* **193**, 1–12 (2015).

4. Scheres, S. H. W. RELION: implementation of a Bayesian approach to cryo-EM structure determination. *J. Struct. Biol.* **180**, 519–30 (2012).

5. Emsley, P., Lohkamp, B., Scott, W. G. & Cowtan, K. Features and development of Coot. *Acta Crystallogr. D* **66**, 486–501 (2010).

6. Liebschner, D. *et al.* Macromolecular structure determination using X-rays, neutrons and electrons: recent developments in Phenix. *Acta Crystallogr. D* **75**, 861–877 (2019).

7. Terwilliger, T. C. *et al.* Iterative model building, structure refinement and density modification with the PHENIX AutoBuild wizard. *Acta Crystallogr. D* **64**, 61–69 (2008).

8. Afonine, P. V. *et al.* Real-space refinement in PHENIX for cryo-EM and crystallography. *Acta Crystallogr. D* **74**, 531–544 (2018).

9. Schrodinger, L. The PyMOL Molecular Graphics System, Version~1.8. (2015).

10. Pettersen, E. F. *et al.* UCSF Chimera--A visualization system for exploratory research and analysis. *J. Comput. Chem.* **25**, 1605–1612 (2004).

Supplementary information, Table S1

Data Collection and Processing		Refinement			
Microscope	Titan Krios		**CALHM5-RUR**	**Apo-CALHM**	**CALHM5-Ca^{2+}**
		Initial model used (PDB code)	*De novo*	CALHM5-RUR	CALHM5-RUR
Voltage (kV)	300kV	Model resolution (Å)	2.60	2.89	2.9
Detector	K3 Summit direct detector	FSC threshold	0.143	0.143	0.143
Magnification	81,000	Model composition			
Pixel size (Å)	1.1	Chains	11	11	11
Number of frames	32	Non-hydrogen atoms	25168	25168	25168
Electron exposure (e–/Å2)	50	Protein residues	3135	3135	3135
Defocus range (μm)	1.2-1.6	CC map vs. model (%)	0.82	0.82	0.81
Symmetry imposed	C11	R.m.s. deviations			
Initial particle images (no.)	2,954,203 (RUR)<br>3,582,769 (apo)<br>2,741,241 (Ca^{2+})	Bond lengths (Å)	0.005	0.004	0.007
Final particle images (no.)	576,663 (RUR)<br>1,099,624(apo)<br>392,239 (Ca^{2+})	Bond angles (°)	0.777	0.710	0.865
Map resolution (Å)	2.60	Validation			
FSC threshold	0.143	MolProbity score	1.17	1.14	1.32
Ligand1	Phosphatidic acid	Clashscore	1.94	1.99	2.82

	Poor rotamer	0.00	0.00	0.00
	Ramachandran plot			
	Favored (%)	96.72	97.01	96.24
	Allowed (%)	3.28	2.99	3.76
	Disallowed (%)	0.00	0.00	0.00

Supplementary information, Figures

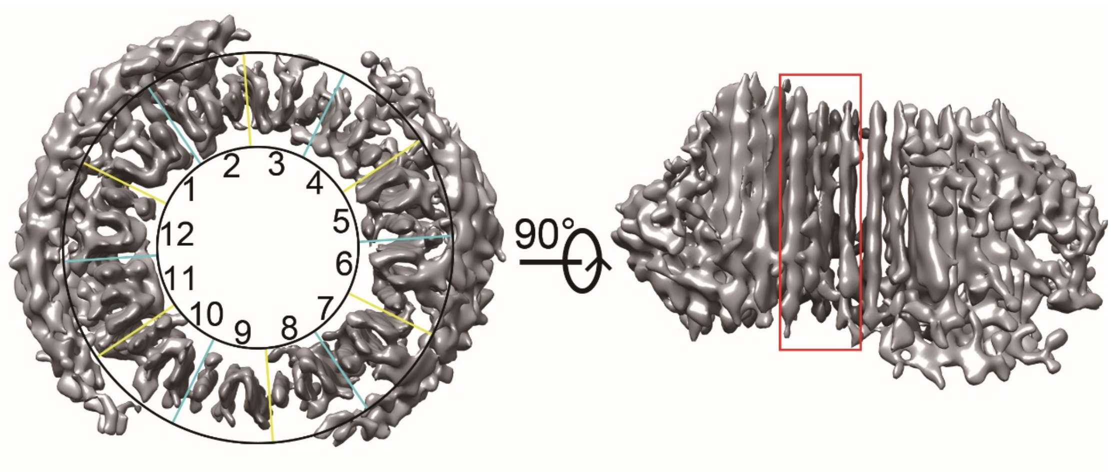

Figure S1. The low-resolution Cryo-EM reconstruction of dodecameric CALHM5 in detergent.

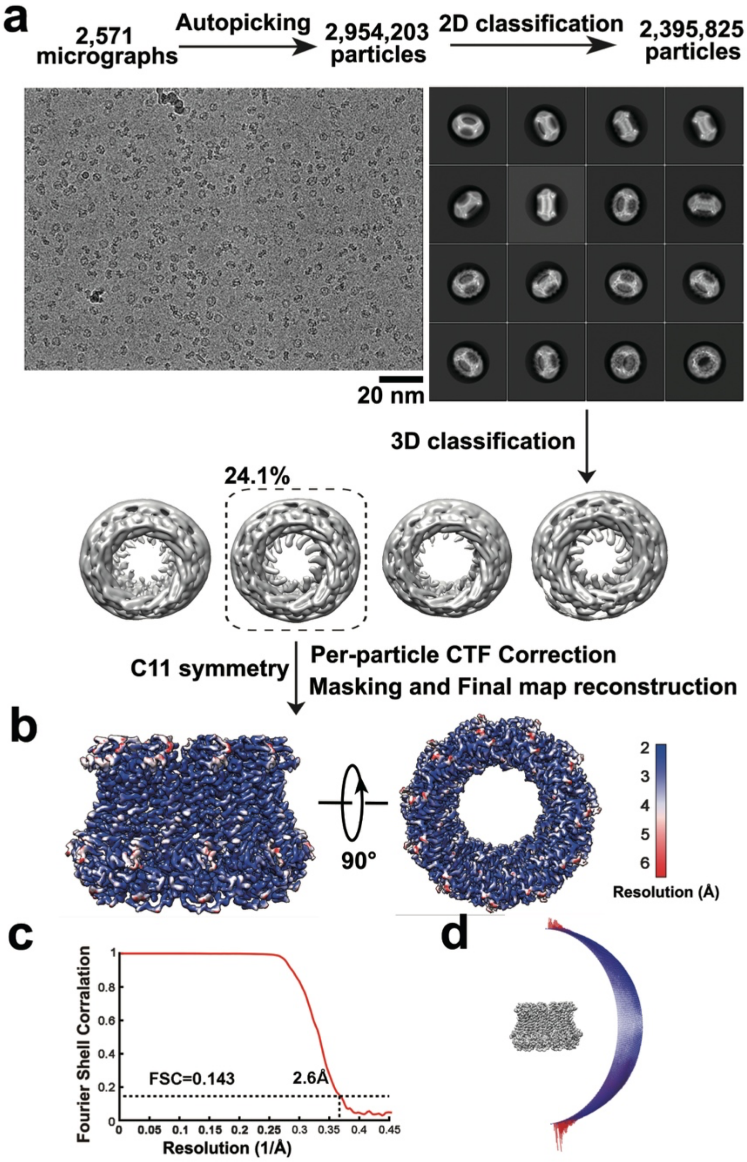

Figure S2. Single particle analysis of CALHM5 with RUR. **a** A representative micrograph, representative 2D and 3D classification averages and reconstitution workflow. **b** The Cryo-EM density map of CALHM5 colored by local resolution. **c** Golden standard FSC curves of the final 3D reconstruction. **d** The angular distribution of particles used for the refinement of CALHM5.

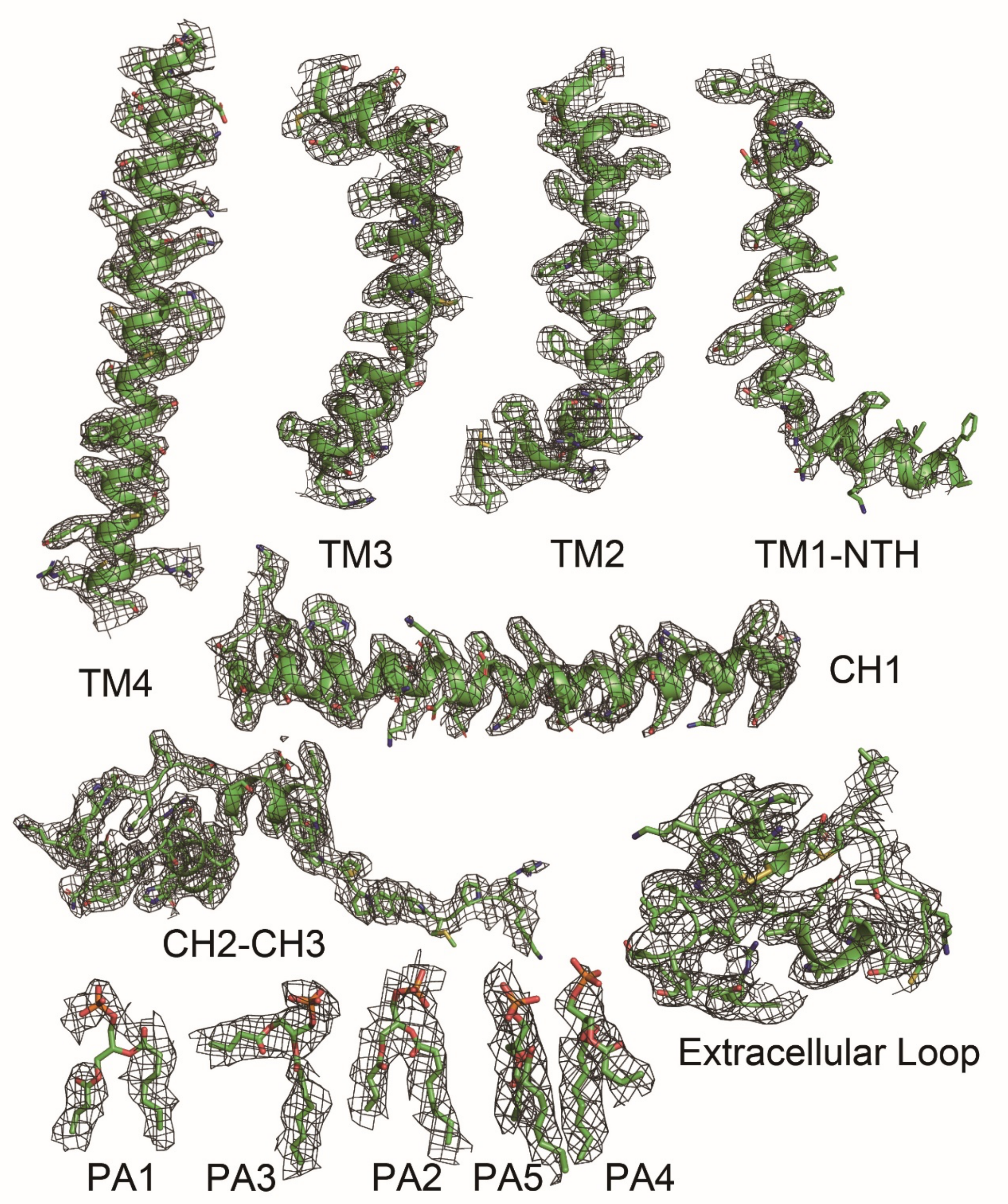

Figure S3. Representation of Cryo-EM density maps of CALHM5.

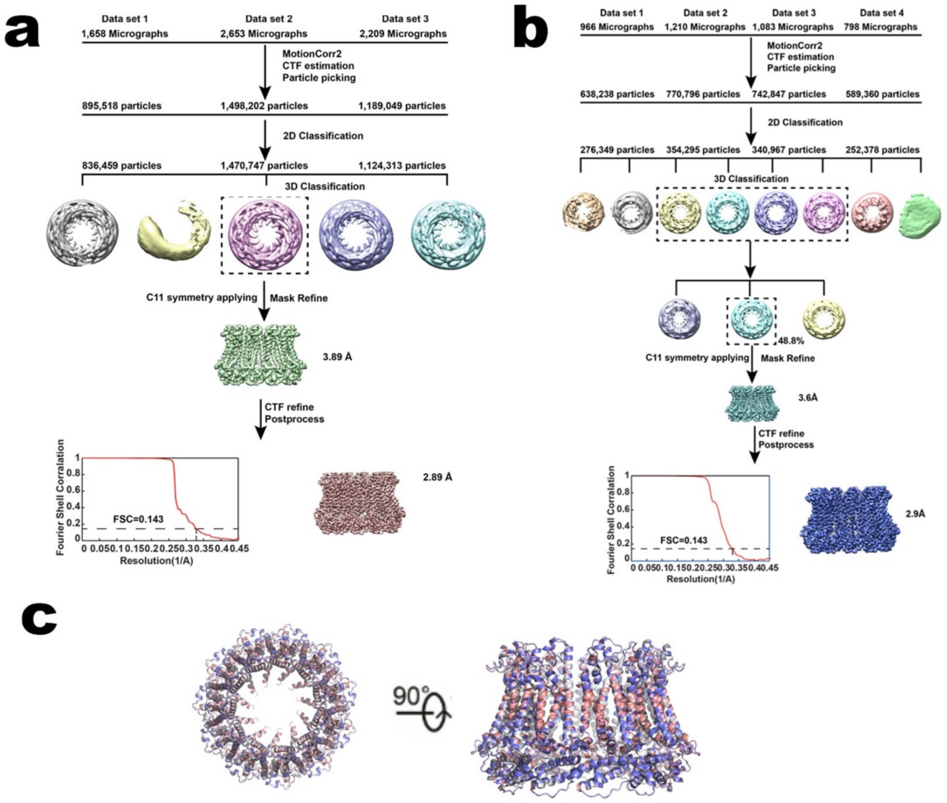

Figure S4. Single particle analysis of CALHM5s and structure alignment. **a** Representative 2D and 3D classification averages and reconstitution workflow of apo-CALHM5. **b** Representative 2D and 3D classification averages and reconstitution workflow of CALHM5 with Ca^{2+}. **c** Structure alignment of CALHM5-Ca^{2+} (gray), apo-CALHM5 (blue), CALHM5-RUR (pink)

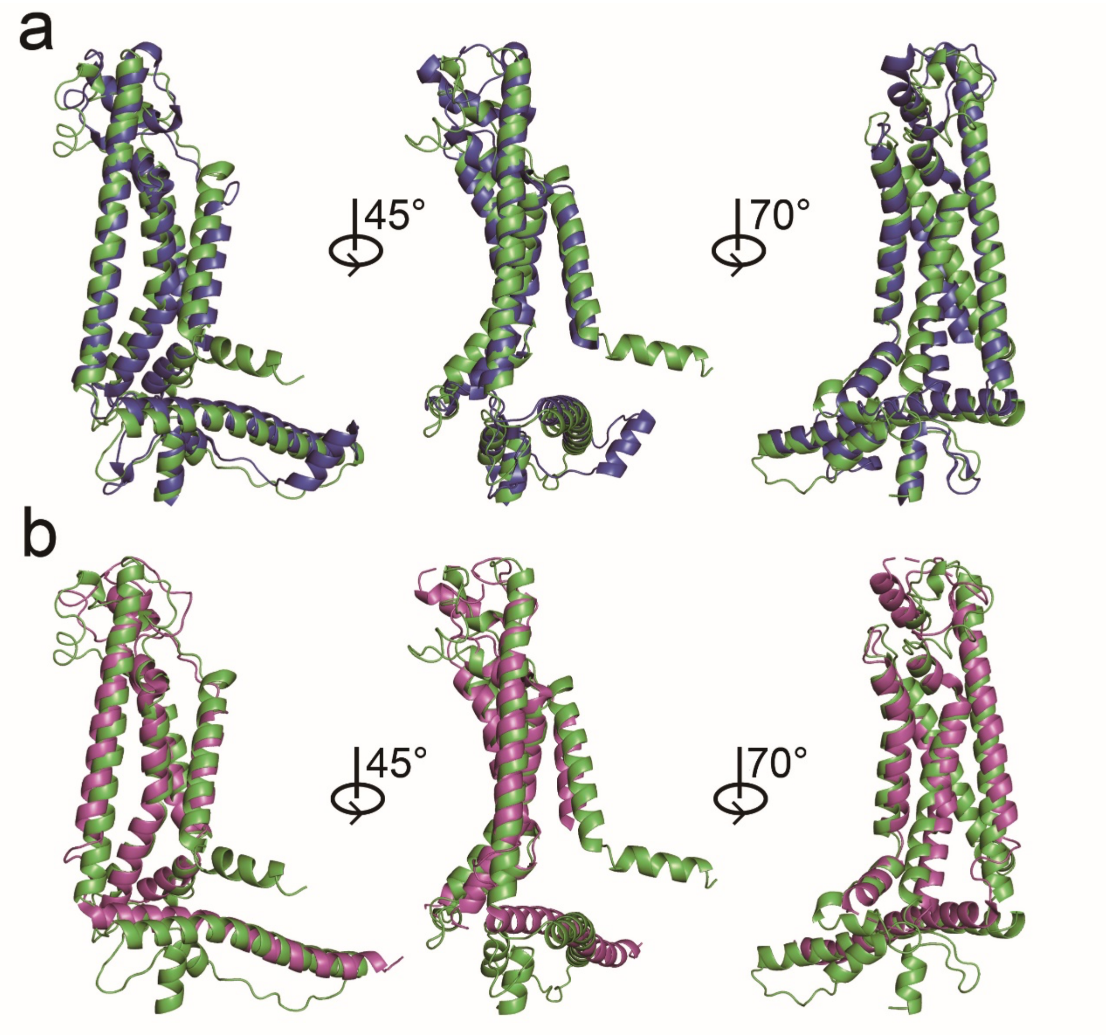

Figure S5. Structure comparisons between CALHM5 and other CALHMs. **a** Representation of a superposition of CALHM5 protomer and hCALHM2 (PDB: 6UIV) protomer. CALHM5 is shown in green and hCALHM2 is shown in blue. **b** Representation of a superposition of CALHM5 protomer and chCALHM1 (PDB: 6VAM) protomer. CALHM5 is shown in green and chCALHM1 is shown in purple.

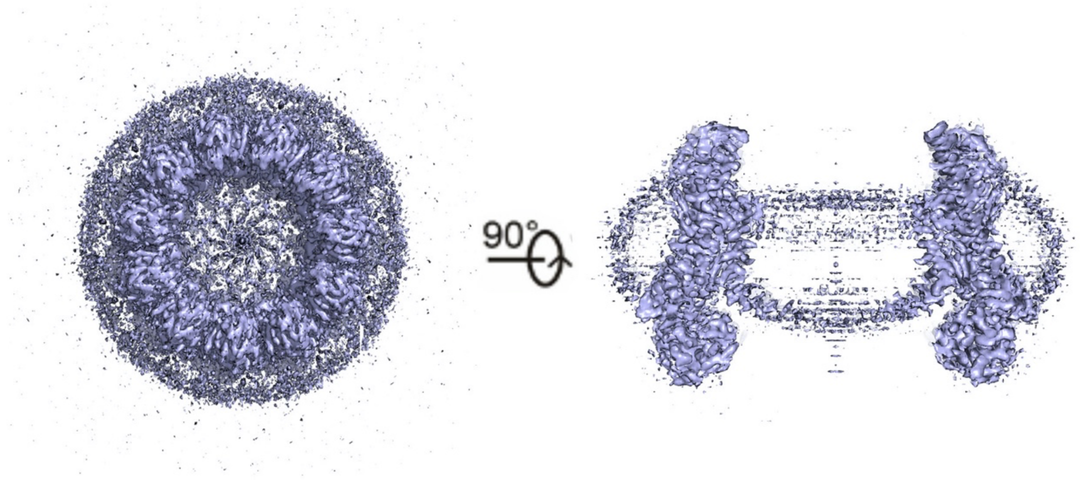

Figure S6. Electron density map of CALHM5-RUR with extra amorphous density in the middle of the pore

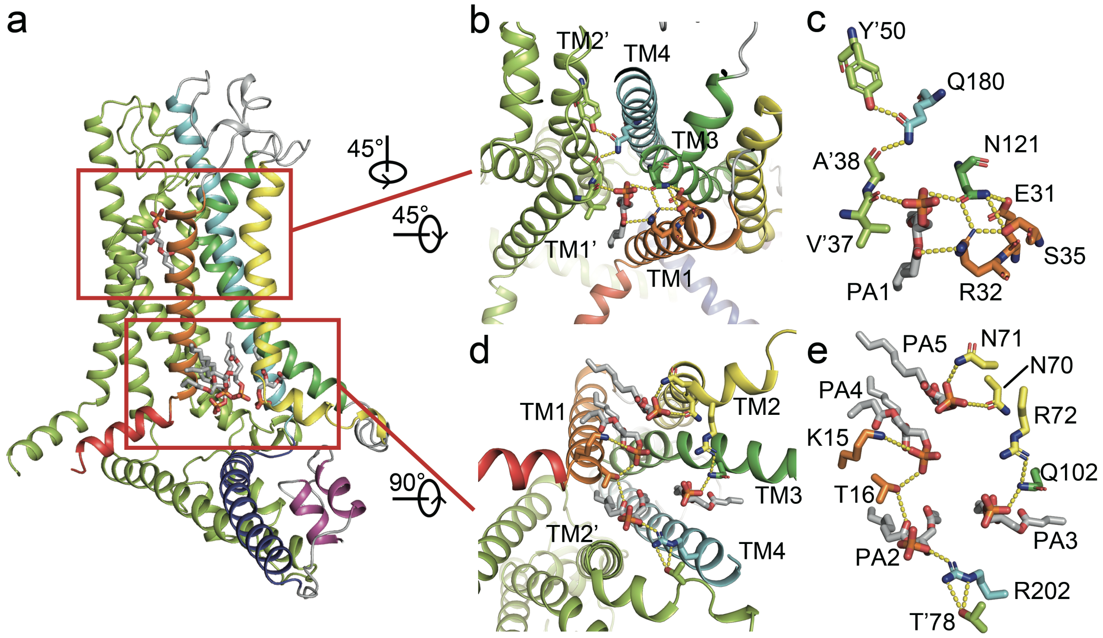

Figure S7. Protein-lipids interactions in CALHM5. **a** Zoom in views of lipid binding sites. Lipid containing subunit is colored same as Figure 1h and adjacent subunit is colored in lemon green. Domains surrounding lipids are labeled (" ' " represent adjacent subunit) with key residues shown in stick. **b,c,d,e** Representation of residues-lipids interaction shown in **a** with all residues labeled.

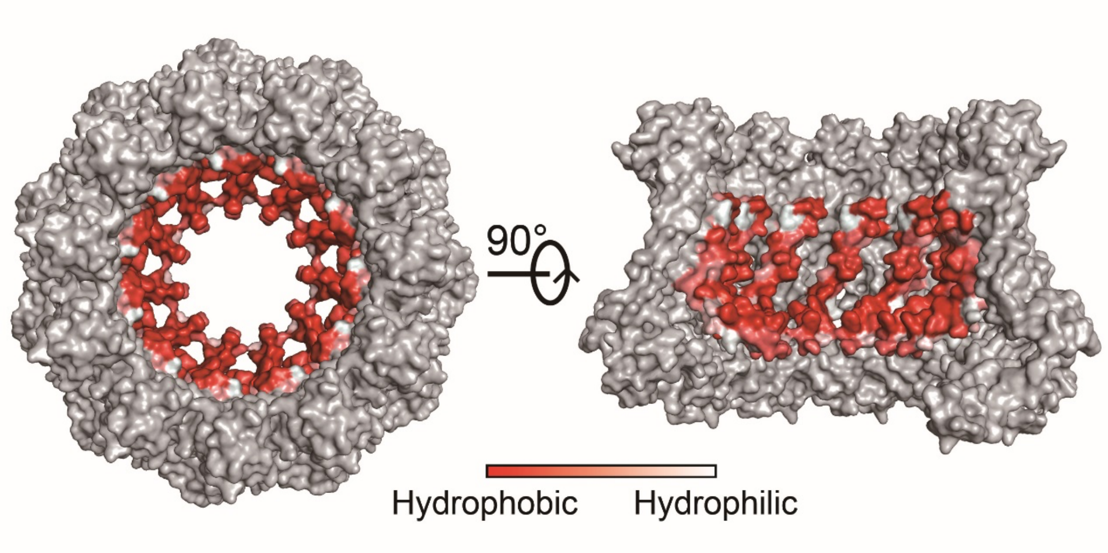

Figure S8. Cartoon representation of channel pore with all residues in NTH and TM1 colored on the basis of relative hydrophobicity.

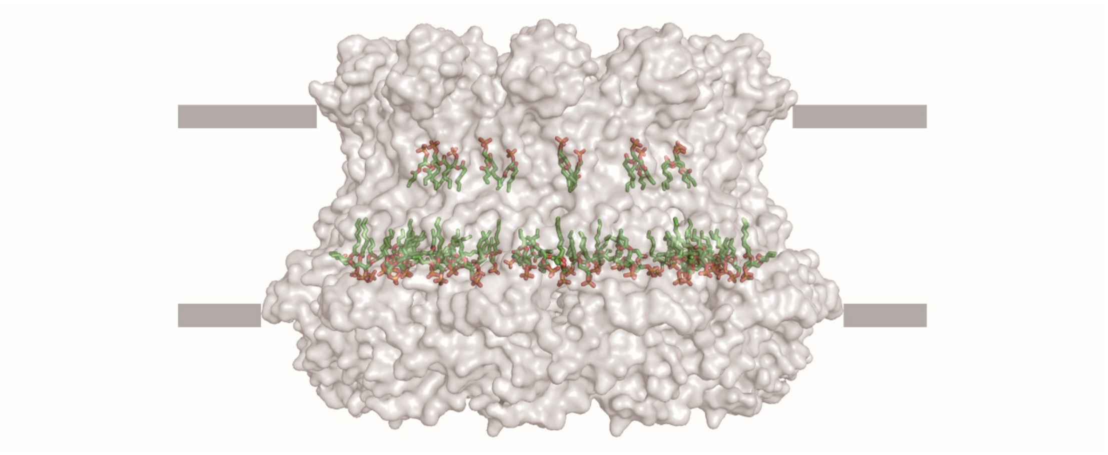

Figure S9. Representation of lipids distribution in CALHM5 channel.

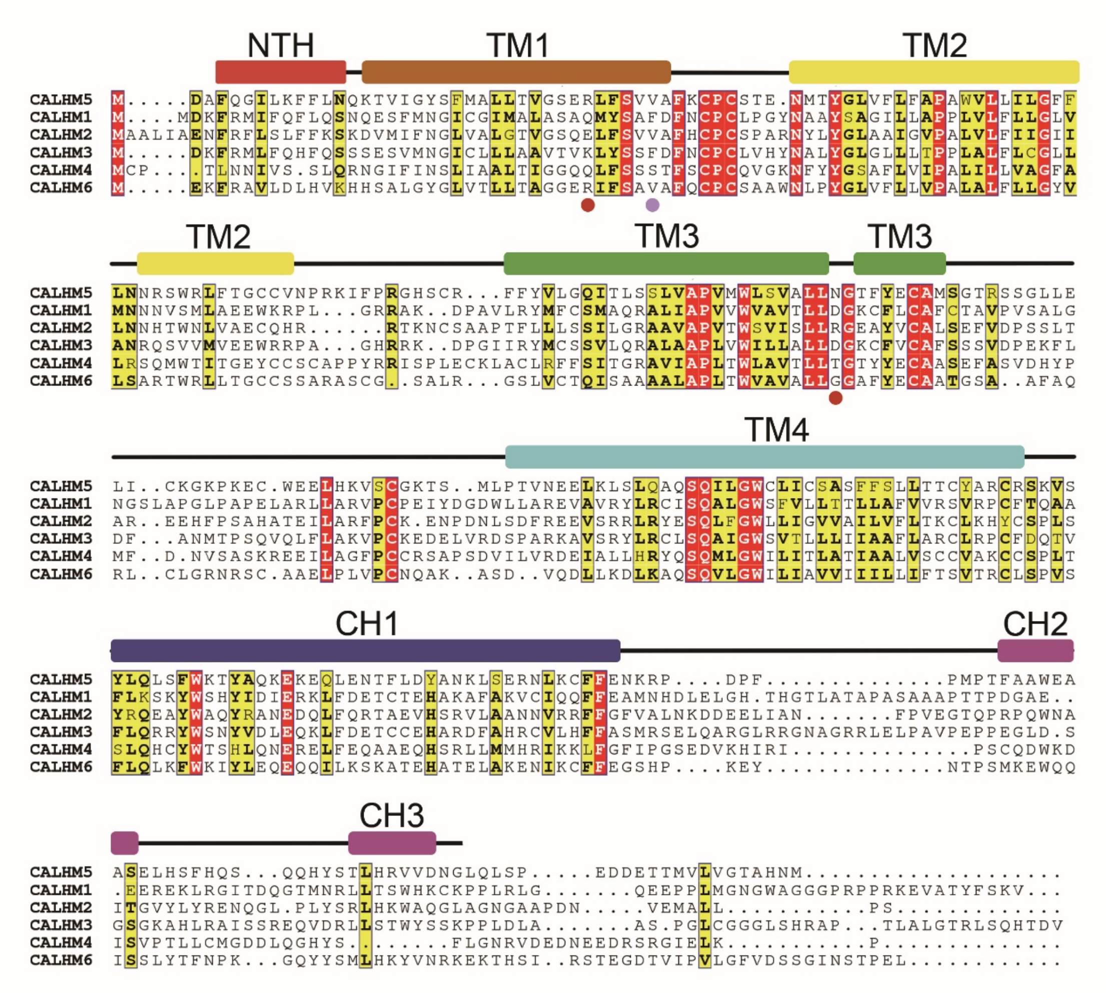

Fig S10. Sequence alignment of human CALHMs. Residues interacting with both PA1 in CALHM5 and RUR in CALHM2 are marked with red circle. Residue interacting with PA1in CALHM5 but not RUR in CALM2 is marked with purple circle.

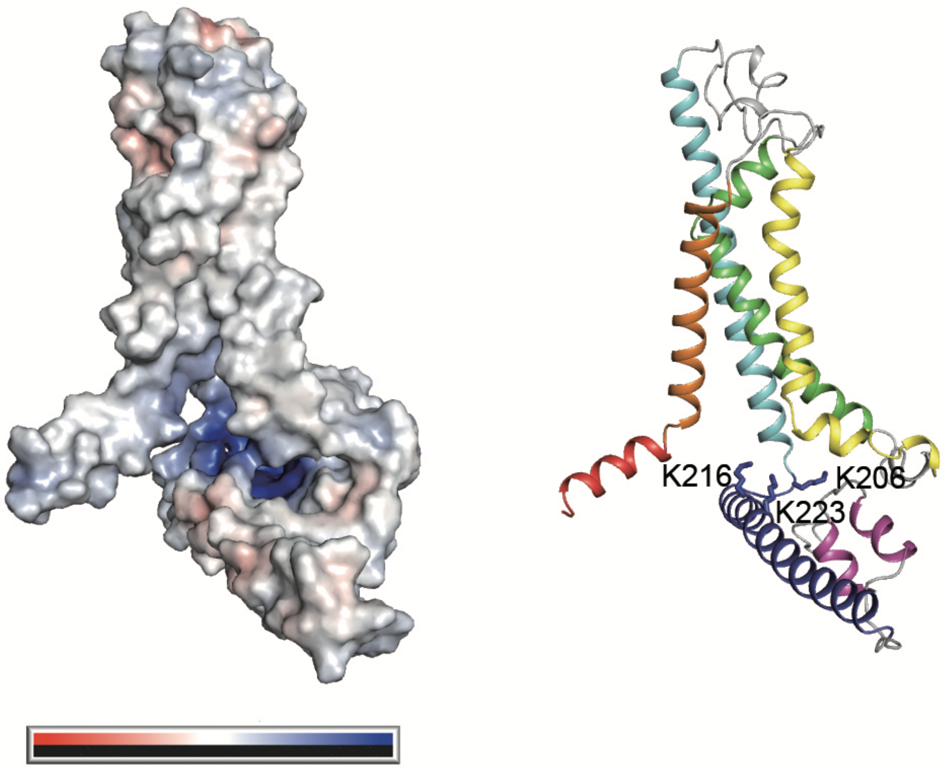

Fig S11. Representation of positively charged cleft with key residues highlighted.
